# Physical Therapy for Knee Pain Relief Induces Changes in Gut Microbiome Composition: A Secondary Analysis of Data From a Randomized Controlled Trial

**DOI:** 10.1177/19417381241283812

**Published:** 2024-10-06

**Authors:** Afroditi Kouraki, Amrita Vijay, Sameer Gohir, Bonnie Millar, Anthony Kelly, Ana M Valdes

**Affiliations:** † Academic Unit of Injury, Recovery and Inflammation Sciences, Rheumatology, School of Medicine, University of Nottingham, Nottingham, UK; ‡ NIHR Nottingham Biomedical Research Centre, Nottingham University Hospitals NHS Trust and the University of Nottingham, Nottingham, UK; § Pain Centre Versus Arthritis, University of Nottingham, Nottingham, UK; ‖ Circle Integrated Care The Barn BMI, Manor Rd, Church End, Bedford, UK

**Keywords:** gut microbiome, muscle strength, physical function, physiotherapy exercise, randomized controlled trial

## Abstract

**Background:**

Aerobic exercise alters gut microbiome composition, yet the impact of gentle physiotherapy on gut microbiome and its relation to muscle strengthening and physical function remains unexplored.

**Hypothesis:**

Physiotherapy exercises modulate gut microbiome composition and changes in gut microbes are linked to improvements in muscle strength or function.

**Study Design:**

Secondary data analysis of samples from a randomized controlled trial.

**Level of Evidence:**

Level 2b.

**Methods:**

Data from a 6-week randomized controlled trial of physiotherapy for knee pain were analyzed. Gut microbiota profiling utilized 16S sequencing. We compared intervention and control (usual care) groups using microbial diversity metrics. Amplicon sequence variants (ASVs) that changed after the program were identified with ALDEX2, and correlations between these ASVs and measures of physical function, muscle strength, and interleukin-6 (IL-6) were explored.

**Results:**

No diversity changes were observed between standard care (n = 43) and physiotherapy (n = 34). Physiotherapy led to significant increases in *Alistipes*, *Bacteroides*, *Clostridium sensu stricto 1*, and *Faecalibacterium* ASVs. Of these, *Clostridium sensu stricto 1* and *Faecalibacterium* were associated with postintervention muscle strength. Increase in *Faecalibacterium* was correlated with a decrease in IL-6 in the physiotherapy group.

**Conclusion:**

Physiotherapy had modest effects on gut microbiome composition affecting 4 taxa. Increases in muscle strength were correlated with increases in 2 taxa including *Faecalibacterium. Faecalibacterium* was also linked to reduced inflammation. Improved walking speed was linked to an increase in *Alistipes* with no differences found for strength or squatting ability.

**Clinical Relevance:**

Improved gut microbiome composition is linked to better overall health outcomes, including enhanced immune function, reduced inflammation, and improved metabolic health. This is particularly relevant for patients with osteoarthritis, who are known to have a high prevalence of cardiometabolic comorbidities. Integrating physiotherapy protocols that positively influence the gut microbiome can thus enhance overall patient outcomes.

Physical exercise has been shown to be an external factor that can change the composition of a person’s gut microbiome yielding health benefits.^38^

Aerobic exercise has indeed been shown in various interventional studies to substantially modify the gut microbiome composition,^3,15,30,41^ resulting in higher abundances of short-chain fatty acid (SCFA) producers.^3^ In addition, a number of large observational studies have shown that physical activity levels are correlated with marked changes in gut microbiome composition.^24,36^ Observational studies have also found that multiple microbiome taxa associated with physical activity are correlated with muscle strength measured by handgrip strength,^24^ indicating that it is not just the aerobic element of physical activity that modulates gut microbiome composition.

A systematic review found that increased physical activity in nonathletes significantly impacted the relative abundance of SCFAs. Aerobic exercise lasting 60 minutes and activities at 60% HRmax or higher also affected beta diversity indexes. Athletes were shown to have a more diverse gut microbiota than nonathletes, but with fewer SCFA- and lactic acid-producing bacteria, suggesting that intense exercise might negatively affect gut microbiota.^16^ Higher alpha diversity in microbiota is not always beneficial and may include harmful bacteria, such as the increased abundance of the potentially pathogenic genus *Veillonella* in marathon runners.^7,23,31^

A prospective metagenomic and metabolomic analysis indicated that exercise-induced improvements in cardiorespiratory fitness and body composition in sedentary adults do not require significant changes in gut microbiota diversity. Instead, these improvements may be linked to moderate changes in microbiome composition.^14^

Most exercise interventions have focused on endurance training and only a few studies have been conducted on resistance training exercises. Muscle strengthening itself through resistance training may be linked to the gut microbiome, potentially due to a gut-muscle axis connection.^2,10^ The well-known health benefits of exercise may be due partly to its positive effects on gut microbiota. However, excessive exercise can negate these benefits by increasing intestinal permeability and oxidative stress, leading to inflammation and a catabolic state that harms skeletal muscle function.^16^

Physical therapy for pain relief focuses on using various techniques and exercises to alleviate pain, improve muscle strength, and enhance overall function.^35^ Programs for knee pain involve resistance training, including isometric closed and open chain exercises.^43^ Closed kinetic chain exercises promote comprehensive muscle activation, joint stability, and functional movement patterns, while open kinetic chain exercises target isolated joint motion and muscle group activation.

The quantity of physical activity involved in physiotherapy is relatively low, with exercises requiring no aerobic exercise and only modest amounts of time per day of resistance training. The effect of such quantifiable and standardized exercise aimed at pain relief on gut microbiome composition has not been tested to date nor has the relationship between muscle strengthening of the target muscles, overall physical function, and gut microbiome composition been assessed. Investigating physiotherapy’s effect on the gut microbiome in clinical populations, and how improved muscle strengthening and physical function may be linked to the gut microbiome, could inform personalized exercise recommendations, optimizing physiotherapy’s health benefits.

Chronic inflammation, influenced directly by the gut microbiome, may limit muscle protein synthesis and training adaptation.^9^ Investigating the links between changes in gut microbiome composition after physiotherapy with changes in systemic inflammation, and particularly interleukin-6 (IL-6), a key player in chronic inflammation,^20^ may help us understand the underlying mechanisms involved.

It has been proposed in the literature that there is a need for studies that can help understand the mechanisms that determine changes in the gut microbiome caused by exercise and their related effects.^38^

The purpose of this study, therefore, was to investigate the effect of a web-based physiotherapy program aimed at reducing pain relief on gut microbiome diversity and composition. This was done by performing secondary data analysis on samples collected as part of a randomized controlled trial that involved participants with knee pain performing approximately 10 to 15 minutes per day of physiotherapy exercises for 6 weeks.^22^ We hypothesized that specific bacteria might be affected by physiotherapy and this would be linked to quadriceps and hamstring muscle strength, improved physical function and levels of inflammation.

## Methods

### Study Population

Data were taken from the randomized controlled trial, iBEAT-OA, testing the effectiveness of the Joint Academy (JA) App in a knee osteoarthritis (OA) cohort of community dwelling persons (age, >45 years).^22^ Participant selection, and inclusion and exclusion criteria have been described in detail previously for the original intervention study. Briefly, participants were included in the trial if they were aged ≥45 years with clinical diagnosis and radiographic confirmation of knee OA (Kellgren and Lawrence grade ≥1). Further inclusion criteria were the ability to read and write English and having access and ability to use a smartphone or tablet. Exclusion criteria were inability to provide consent, terminal or mental illness, neurological conditions, inflammatory joint diseases, dementia, diagnosed sleep apnea, recent acute knee injury, unstable heart condition, rapid hypertension fluctuations, or a body mass index (BMI) >50.

A detailed flow diagram of participant recruitment, group allocation, the selection of participants included in the present secondary analysis (i.e., those with available gut microbiome composition), and the number of observations used for each analysis is outlined in Appendix Figure A1 (available in the online version of this article).

All participants provided written informed consent. Ethical approval was obtained from the Research Ethics Committee (reference No. 18/EM/0154) and the Health Research Authority (protocol No. 18021). The trial is registered under the clinicaltrials.gov database.

### Physiotherapy Intervention

We used the app-based knee exercises developed by JA—a web-based program derived from a physiotherapy exercise program tested widely in Sweden, known as the “Better Management of Patients with Osteoarthritis” (BOA),^53^ i.e., a digital version of the face-to-face program. This intervention has been rated good to very good by 94% of patients at reducing pain related to knee OA and has an excellent safety profile and acceptability.

The exercise program incorporates a variety of movements, including both open and closed chain exercises, as well as a mix of concentric and eccentric exercise techniques. It primarily targets the overall strength of the leg muscles, including those around the hips and knee joints. Open kinetic chain exercises involve a sequence of joints where the end segments can move freely, whereas closed chain exercises involve fixing the far end of the extremity to a stationary object while allowing movement at proximal joints.^4^ The program also includes exercises to enhance balance and provides educational sessions covering the fundamentals of OA, its treatment, self-management of OA symptoms, and the advantages of maintaining a healthy lifestyle.

### Muscle Strength and Physical Function

Isokinetic peak torque of quadriceps and hamstring muscles was measured as Newton meters (Nm) at 60 deg/s and 180 deg/s using a HUMAC / NORM Testing and Rehabilitation System model 7709 (Computer Sports Medicine). A standardized protocol was used and is a valid and reliable assessment method of muscle strength in quadriceps and hamstrings.^33,52^ Specifically, each participant completed 3 repetitions of maximal voluntary isokinetic knee extensions with the affected leg before and after the intervention. There was a 30-second rest between each test and a 1-minute break between velocity changes. The maximum torque limit was set at 300 Nm with a sample frequency of 200 Hz. To ensure standardized testing, subjects were provided with clear instruction and feedback, verbal encouragement, and were allowed to reject nonmaximal efforts. Participants were positioned upright with the Cybex dynamometer and chair according to manufacturer specifications, minimizing extraneous movements, and maintaining a constant hip joint angle of 90°. The resistance pad was placed at the ankle joint, and subjects were secured at the thigh, waist, and chest with arms folded across the chest and an emergency stopper in hand. The best peak torque of the 3 contractions was recorded for each angular velocity.

The 30-second sit-to-stand test (30-CST) was assessed as the number of times the participant could rise from a sitting position on a chair to a full standing position in 30 seconds. At enrollment, participants had a demonstration of the test and practiced once before doing the test. The 30-CST was conducted once to avoid fatigue. The timed up-and-go (TUG) test was assessed as the time (measured in seconds) required for the participant to stand up on therapist’s command, walk 3 meters, turn around, walk back to the chair, and sit down again. The TUG test was conducted 3 times and the mean time was used; 30-CST and TUG are validated methods used to assess participants’ lower body strength and functional mobility, respectively.^28,46^

### Stool Sample Collection

Participants were given a stool collection kit and leaflet with detailed instructions on how to collect and post the sample to the assessment center in the prepaid postbox provided at the end of their baseline and follow-up visits. All samples were either immediately frozen at -80°C or at -20°C temporarily until they could be transported to -80°C freezers at the hospital following local standard operating procedures (SOPs) before analysis. SOPs were used at the collection sites and when transferring the samples, which were handled by trained research personnel to ensure the high quality and reliability of the research data. Stool samples were outsourced to an external supplier for DNA extraction, quality control, and preprocessing (see next section).

### Bioinformatics

The wet-laboratory procedure was conducted by the Bioinformatic Genetic Lab, Department of Internal Medicine Erasmus Medical Center, Rotterdam. Stool DNA extraction was carried out using the DNA isolation kit Invimag Stool DNA Kit by Stratec and 100 mg of stool sample. Gut microbiome composition was determined by 16S rRNA gene sequencing carried out on a Illumina MiSeq, using V3 chemistry, at 2 × 300 bp. Quality control (QC) and preprocessing including demultiplexing using QIIME1 Version 1.9.1 and primer trimming with TagCleaner Version 0.16 were performed by the Bioinformatic Genetic Laboratory. Reads were exported from the MiSeq as a run level FASTQ file, containing all reads generated above Q30, the mean QC value threshold used by Illumina. Briefly, >Q30 means that each nucleotide in the read has at maximum a 1:1000 chance of being called wrong. The DADA2 pipeline in R Version 4.0.0 was used for analysis of the sequencing data that turns sample-wise amplicon sequences into a feature count.^11^ Amplicon sequence variants (ASVs) from DADA2 infer real sample sequence variants within a sample, and represent true, observed amplicon sequence fragments from samples.

### Statistical Analysis

We carried out all statistical analyses in R Version 4.3.0. Differences between the descriptive characteristics of the control and intervention groups were evaluated used Student *t* tests or Mann-Whitney U tests for continuous variables and Chi-squared tests for categorical variables.

ASVs with a relative abundance of <0.01% in every sample were removed using a function adapted from Arumugam et al.^5^ Filtering resulted in 686 ASVs (from 1833) remaining for analysis. Taxonomical results were analyzed only at genus taxonomic levels. The Shannon index was calculated using the vegan package. For beta diversity for community composition and functional capacity over time, we used the Bray-Curtis (BC) dissimilarity and principal coordinate analysis. The permutational multivariate analysis of variance (PERMANOVA) test was used to test differences in BC between control and intervention groups. An overview of the PERMANOVA method and more details on its use in the context of the present analysis are provided in the Online Appendix.

We used the ANOVA-like differential expression (ALDEX2) tool. This is a method used commonly in microbiome studies for analyzing differential abundance of microbial taxa designed to identify microbial taxa that are differentially abundant between different groups or conditions in a microbiome dataset and has been shown to be very consistent in replicating results across different datasets.^42^ An overview of the ALDEX2 method and the steps it performs is provided in the Online Appendix.

We used this method to investigate changes in the most abundant ASVs in the intervention group from baseline to follow-up. The most abundant ASVs were defined as having >1% mean relative abundance (as previously defined in the literature and being present in ≥50% of the investigated subjects),^15,25,57^ which limited the number of ASVs included in analysis to n = 18. We controlled for false discovery rate (FDR) due to multiple testing with the Benjamini-Hochberg (B-H) method.^8^

We then used ADLEX2 to identify differences in ASVs between those who improved their quadriceps and hamstring muscle strength, 30-CST, and TUG after the physiotherapy intervention. For TUG, improvement was defined as a decrease of ≥0.5 seconds in the time taken to complete the test. Similarly, a definition of improvement by ≥0.5 Nm of change in strength was used for quadriceps and hamstring muscle strength and an improvement by ≥1 change in 30-CST measured as number of times the participant completed a chair-stand in 30 seconds. Details on the use of ALDEX2 in the context of the present analysis are provided in the Online Appendix. The FDR B-H method was used to control for multiple testing.

## Results

### Descriptive Characteristics, Isokinetic, and Physical Function Measures

The descriptive characteristics of the entire sample and of the 2 subgroups (control arm and intervention arm) are presented in Table 1. The 2 arms did not differ in any descriptive characteristics studied (e.g., age, sex, BMI). As previously described,^21^ significantly greater increases in all measures of physical function and muscle strength and a trend of increase for quadriceps strength at 180 deg/s were observed in the physiotherapy arm, and these were significantly greater than in the control arm in this subsample.

**Table 1.** Baseline demographics (sex, age, and BMI) as well as baseline and follow-up muscle strength (isokinetic PT) and physical function measures (TUG and 30CST) of the entire sample and of the 2 subgroups (control and intervention arm)^*a*^

Feature	All			Control			Intervention		*P*
Participants, n (%)	77			43			34		
Sex, n (%)									
Female	52 (67.53)			28 (65.11)			24 (70.59)		0.61
Age, y	68.55 (8.71)			70.30 (7.71)			66.40 (9.48)		0.07
BMI, kg/m^2^	30.81 (5.44)			31.72 (5.62)			29.69 (5.07)		0.10
	**Baseline**	**Follow-up**	*P*	**Baseline**	Follow-up	*P*	**Baseline**	Follow-up	*P*
30CST^*b*^	8.94 (3.41)	11.54 (5.26)	<0.01	8.58 (3.94)	9.70 (4.76)	<0.01	9.37 (2.61)	13.80 (5.01)	<0.01
TUG, seconds^*c*^	9.97 (2.97)	9.47 (3.69)	<0.01	10.75 (3.64)	10.48 (4.48)	.02	9.03 (1.42)	8.23 (1.77)	<0.01
Isokinetic PT, Nm^*d*^									
	75.49 (45.12)	82.06 (46.81)	<0.01	70.60 (42.10)	75.74 (42.73)	.04	81.49 (48.51)	89.83 (50.94)	<0.01
	53.49 (27.84)	61.31 (30.94)	<0.01	52.81 (26.08)	57.70 (29.31)	.01	54.31 (30.23)	65.74 (32.72)	<0.01
	43.79 (26.80)	48.71 (29.98)	0.02	41.26 (24.41)	45.26 (30.34)	.15	46.91 (29.53)	52.94 (29.41)	0.06
	39.37 (21.60)	44.91 (24.56)	<0.01	38.63 (21.77)	43.37 (25.31)	.03	40.29 (21.67)	46.80 (23.83)	<0.01

BMI, body mass index; 30CST, 30-second sit-to-stands; PT, peak torque; TUG, timed up-and-go.

a Data are expressed as mean ± SD unless otherwise stated.

b Counts the number of times the participant comes from a sitting position on a chair to a full standing position in 30 seconds.

c Measured in seconds, the participant stands up at therapist’s command, walks 3 meters, turns around, walks back to the chair and sits down.

d Isokinetic peak torque (Nm) of quadriceps and hamstring muscles measured at 60 deg/s and at 180 deg/s.

*P* values from comparisons between groups used Student *t* tests or Mann-Whitney U tests for continuous variables and Chi-squared tests for categorical variables.

### No Changes Observed in Alpha and Beta Diversity in Response to Physiotherapy

**Figure 1.**
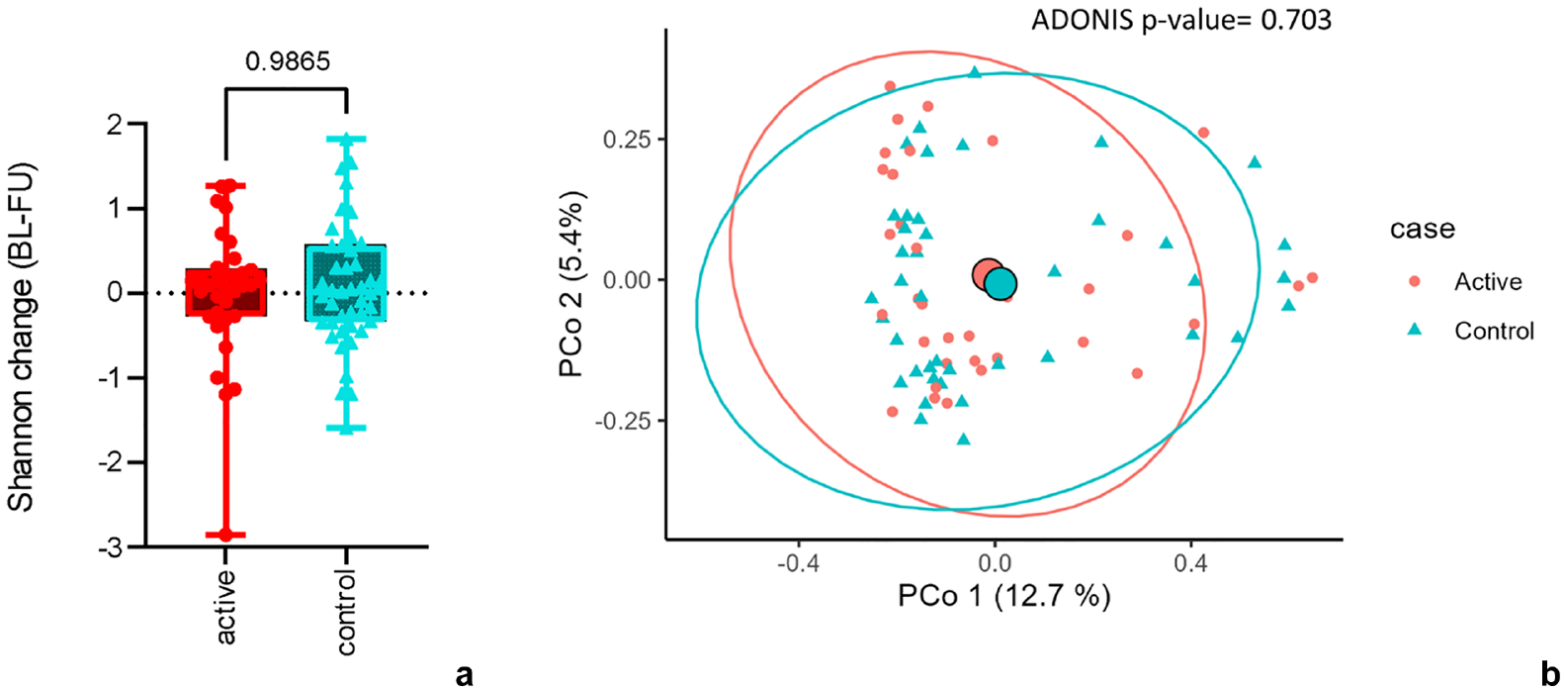
Comparisons of change in α diversity (Shannon index) and β diversity between active (physiotherapy) and control groups. (a) Boxplots of change in Shannon from baseline to follow-up in active (red) and control (light blue) conditions. (b) PCoA of Bray-Curtis distance of change from BL-FU, visualizing microbial composition in active (red) and control (light blue). BL-FU, baseline to follow-up; PCoA, principal coordinate analysis.

We first examined changes in alpha and beta diversity between the physiotherapy and control arms. No difference was observed between arms in the change of Shannon index from baseline to follow-up (*P* > 0.05) (Figure 1a). Similarly, no difference in beta diversity was observed in the change data from baseline to follow-up between the active and control groups (*P* > 0.05) (Figure 1b).

### Changes in Gut Microbiome Composition in Response to Physiotherapy

**Figure 2.**
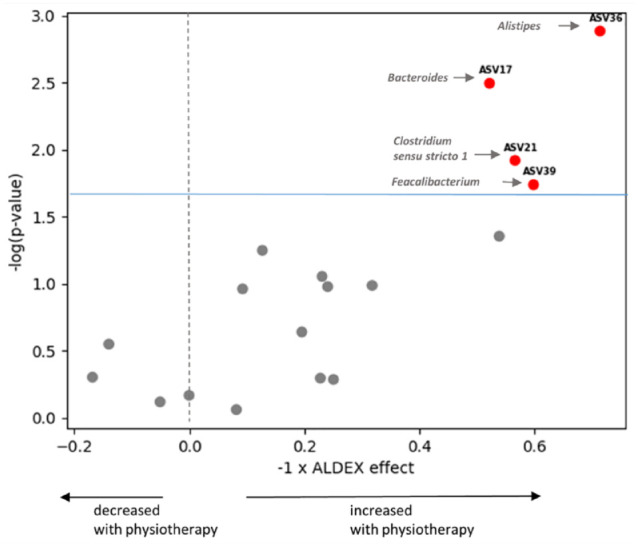
Volcano plot of ASVs that changed after physiotherapy. Annotated ASVs marked in red passed FDR threshold of <10% (light blue horizontal line) and were increased with physiotherapy. ASV, amplicon sequence variant; FDR, false discovery rate.

When we tested for differential abundance, however, we found that physiotherapy led to increases in the abundance of 4 ASVs belonging to the genera *Alistipes*, *Bacteroides*, *Clostridium sensu stricto 1*, and *Faecalibacterium* (Figure 2) which achieved an FDR of <10%. *Alistipes* achieved an FDR of <5%.

Since these changes may be reflecting higher levels of physical activity in the physiotherapy group, due to increased muscle strength, we tested whether these microbial taxa were also correlated with changes in quadriceps and hamstring muscle strength in response to physiotherapy.

### Genera That Increased in Response to Physiotherapy Correlate With Muscle Strength

**Figure 3.**
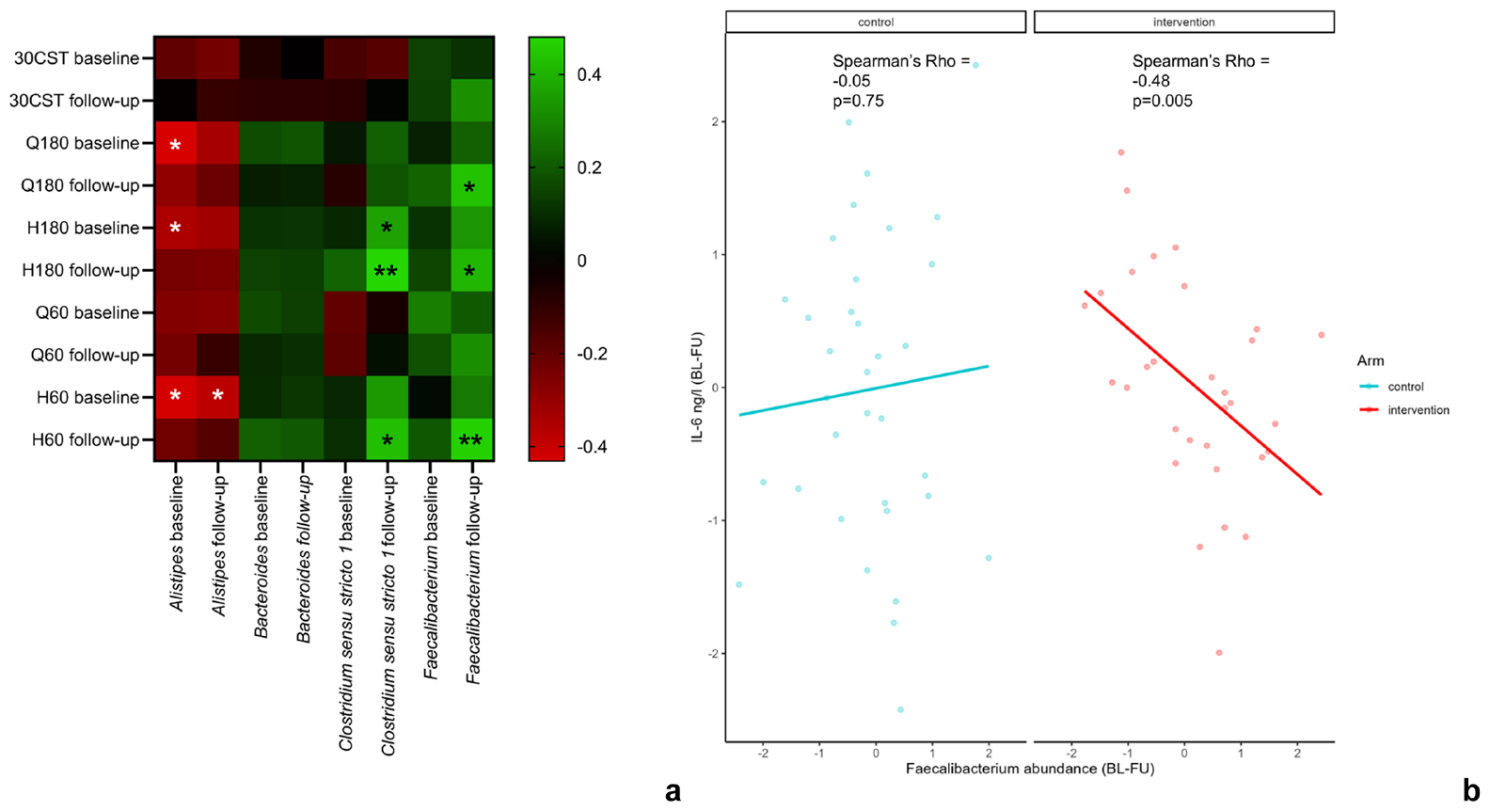
(a) Heatmap plot of correlations between the ASVs that increased with physiotherapy and isokinetic muscle strength and 30-CST measures at baseline and follow-up in the physiotherapy group (*P < 0.05 and **P < 0.01). (b) Scatterplots of the correlation between BL-FU change in IL-6 (ng/l) and change in *Faecalibacterium* ASV abundance in active (red) and control (light blue) groups separately. ASV, amplicon sequence variant; BL-FU, baseline to follow-up; 30-CST, 30-second sit-to-stand test; H, hamstring; IL-6, interleukin-6; Q, quadriceps.

The *Alistipes* ASV that increased in response to physiotherapy exercise was found negatively correlated with hamstring strength (both at 60 deg/s and at 180 deg/s) and quadriceps strength (180 deg/s) at baseline but not at follow-up in the intervention group (Figure 3a). The *Clostridium sensu stricto 1* and *Faecalibacterium* ASVs, both of which increased in response to physiotherapy were correlated positively with hamstring strength at 60 deg/s and 180 deg/s at follow-up. *Faecalibacterium* ASV was further positively correlated with quadriceps strength (180 deg/s) at follow-up. This may suggest that these 2 ASVs change in response to muscle strengthening from physiotherapy exercise. None of the ASVs that increased with physiotherapy were correlated with the ability to perform chair-stands (30-CST).

### Increase in *Faecalibacterium* Correlates With Decrease in Proinflammatory Marker IL-6

We next explored whether the proinflammatory marker, IL-6, is linked to any of the 4 ASVs that were increased with physiotherapy. Increase in *Faecalibacterium* ASV from baseline to follow-up was correlated strongly with a decrease in IL-6 only in the intervention group (*P* < 0.01). No correlation was found between change in IL-6 and change in this *Faecalibacterium* ASV in the control group (Figure 3b), and none of the other ASVs were significantly correlated with IL-6.

### *Alistipes* Was Increased in Participants Who Improved Their Walking Speed

**Figure 4.**
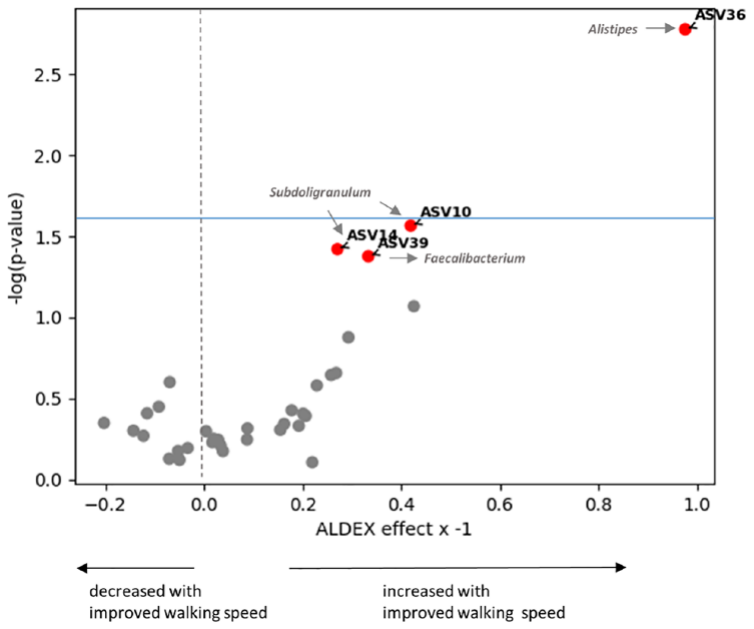
Volcano plot of ASVs that changed after physiotherapy in those who improved their walking speed (TUG). Annotated ASVs marked in red increased in those who improved their walking speed. *Alistipes* (ASV36) passed FDR threshold of <10% (above the light blue horizontal line), whereas the other 3 ASVs were nominally increased (below the light blue horizontal line). ASV, amplicon sequence variant; FDR, false discovery rate; TUG, timed up-and-go.

We next explored whether gut microbiome composition changed after the physiotherapy exercise in those who improved in physical function, i.e., walking speed and ability to perform chair-stands (30-CST), and muscle strength compared with those who did not. Using ALDEX, we compared the ASV abundances between participants whose TUG test had improved by ≥0.5 second (n = 21) with those whose TUG test had not improved (n = 12). We found that the same *Alistipes* ASV that was increased in response to physiotherapy was increased in those who improved their walking speed after physiotherapy (Figure 4). None of the other ASVs reached FDR statistical significance. However, 3 ASVs, 2 belonging to genus *Subdoligranum* and 1 belonging to genus *Faecalibacterium* (the same ASV as the one that increased with physiotherapy), increased in those with improved walking speed, although with nominal significance. No ASV changed with improved squatting ability (30-CST) at FDR <10% or with improvements in quadriceps or hamstring muscle strength (data not shown).

## Discussion

In this study, we investigated the effect of physical therapy on gut microbiome composition and the links between the bacterial taxa modified by physiotherapy and changes in the muscles specifically targeted by physiotherapy, as well as physical function and systemic inflammation. We report that there is no overall effect on gut microbiome diversity or composition induced by physiotherapy for knee pain, compared with a control arm, consistent with previous studies on resistance training. We did find, however, that there are certain bacterial taxa (ASVs) that increased in response to physiotherapy, compared with baseline, among persons who completed the physiotherapy course and among those who improved their walking speed.

Improved gut microbiome composition is linked to better overall health outcomes, including enhanced immune function, reduced inflammation, and improved metabolic health. This is particularly relevant for patients with OA, as in our case, who are known to have a high prevalence of cardiometabolic comorbidities, such as metabolic syndrome and stroke.^29,50^ Integrating physiotherapy protocols that influence the gut microbiome positively can thus enhance overall patient outcomes.

Consistent with our findings, a systematic review of human studies found that exercise interventions had little or no effect on bacterial alpha and beta diversities. Contrasting findings were detected regarding changes in the abundance of *Firmicutes*, *Bacteroidetes*, and *Proteobacteria* in response to exercise.^44^ Unlike the animal studies demonstrating substantial gut microbiome alterations,^12,17^ none of the five human resistance training interventional studies published to date reported significant changes in bacterial composition or overall diversity.^1,10,37,39,41^ However, two studies suggested a potential gut-muscle axis connection, indicating that resistance training might have indirect effects on the gut microbiome through its impact on muscle function.^2,10^ In our study, we observed significant changes in gut microbiome composition after a 6-week course of physiotherapy. Consistent with findings in a murine model of resistance training,^17^ we identified an increase in an ASV belonging to the genus *Alistipes* in response to training. This same ASV was also increased among persons whose walking speed improved with physiotherapy, highlighting *Alistipes* relevance in response to such treatment.

The links in the literature between *Alistipes*, physical activity, and muscle strength are controversial. *Alistipes* has been previously negatively associated with muscle loss. Tichinesi et al^54^ found higher abundance of *Alistipes shahii* in nonsarcopenic subjects compared with sarcopenic subjects. In contrast, the abundance of the genus *Alistipes* was associated with lower muscle mass in unhealthy older persons.^13^ Furthermore, *Alistipes* is associated with higher risk of hypertension and colorectal cancer,^45^ but it is also associated with promoting healthy phenotypes, such as its protective roles in conditions like colitis, autism spectrum disorder, and various liver and cardiovascular fibrotic disorders.^45^ Importantly, in a recent analysis of a subcohort of 307 healthy men, *Alistipes putredinis* was found to modify the association between physical activity and weight loss, with a higher abundance of *A. putredinis* resulting in greater weight loss in response to physical activity.^56^ The genus *Alistipes* has a high number of protein fermentation pathways among commensal bacteria^45^ and can transform dietary fiber into the antioxidant ferulic acid.^47^ Taken together, these data suggest that *Alistipes*, due to its unique functional properties, has important physiological roles, including potential involvement in response to resistance training.

We also found that ASVs belonging to the genera *Bacteroides*, *Clostridium sensu stricto 1*, and *Faecalibacterium* were increased with physiotherapy. *Bacteroides* spp. play a key role in sustaining the microbial food web^58^ and are principal synthesizers of Vitamin K,^55^ which may prevent or treat osteoporosis by increasing bone mineral density.^19^ The relative abundance of *Bacteroides* was increased significantly in persons undergoing aerobic exercise whereas the relative abundance of *Clostridium* cluster IX (a different cluster to ours) was increased in the trunk training exercise group.^40^

We find a positive correlation between increases in the same *Faecalibacterium* and *Clostridium sensu stricto 1* ASVs that were increased with physiotherapy and measures of quadriceps and hamstring muscle strength after physiotherapy. Consistent with this, *Faecalibacterium* and *Clostridium* have been reported to be increased in bodybuilders compared with long-distance runners and sedentary controls in an observational study.^27^ The result with *Faecalibacterium* was not replicated in a separate observational study in bodybuilders,^51^ possibly because the latter study utilized PCR primers to access counts of selected spp. including *Faecalibacterium prausnitzi*, rather than 16S sequencing as in our study and in the study by Jang et al.^27^

*Faecalibacterium* is a well-known butyrate acid producer linked to anti-inflammatory phenotypes.^32,48^ We find here a strong negative correlation between change, from baseline to follow-up, in the same *Faecalibacterium* ASV that increased with physiotherapy and change in proinflammatory cytokine, IL-6 only in the intervention arm, which highlights the beneficial systemic effects of physiotherapy. Butyrate has a role as an anti-inflammatory agent, primarily via inhibition of nuclear factor κB (NF-κB) activation in human colonic epithelial cells.^26^ NF-κB regulates many cellular genes involved in early immune inflammatory responses, including IL-6.^6^

This same *Faecalibacterium* ASV increased, despite nominally, in those who improved their walking speed after the physiotherapy intervention, consistent with a previous observation from an aerobic exercise intervention in lean compared with obese subjects.^3^ Two ASVs belonging to *Subdoligranulum* genus were also increased nominally in those who improved their walking speed. *Subdoligranulum* is another well-known butyrate acid producer and has been associated with various health benefits.^49^ For instance, *Subdoligranulum* was depleted in patients with type 2 diabetes mellitus with the antidiabetic drugs metformin and acarbose shown to increase its relative abundance^18,59^ and was underrepresented in nonalcoholic fatty liver disease patients.^34^

Overall, we find that the effects of physiotherapy on gut microbiome composition are fairly modest and much less dramatic than those repeatedly found for aerobic exercise.^15,30,41^ Interestingly, we did not find any significant changes in gut microbiome that correlated with improvements in squatting (chair stands), but only with TUG, which is a measure of walking speed, i.e., linked to aerobic exercise and physical activity. This is in agreement with the stronger links between the gut microbiome, aerobic exercise and physical activity already reported and the weak links with resistance training.

We note several limitations of the current study, not least is the very small sample size of both arms of the study. In spite of this, the sample sizes for both arms in this randomized control trial are larger than those reported to date for resistance training.^1,10,37,39,41^ Another important limitation is the lack of metagenomic data, and the availability of only 16S data, i.e., DNA sequence data of bacteria that covers taxonomy reliably only up to the genus and is not informative of specific molecular pathways. In addition, we did not monitor the participants’ diets, which could be a confounding variable potentially partly explaining the microbiome changes observed in our study. However, there were no statistically significant differences in BMI between the control and intervention groups, nor were there significant differences in alpha and beta diversity from baseline to follow-up in the intervention group (Online Appendix Figure A2), which may suggest that there were no substantial shifts in daily eating habits. To better isolate the cause of microbiome changes, future studies should incorporate food frequency questionnaires despite implementation challenges in volunteer-based studies due to the time required for completion. Notwithstanding, we have been able to identify some taxa linked to response to physiotherapy that are consistent with what has been reported in animal models (*Alistipes*) and in aerobic exercise (*Faecalibacterium*). Finally, the study was carried out in persons with painful knee OA; therefore, results may not be generalizable to the general population.

The study also has some significant strengths. We used an analysis pipeline that has been widely validated (ALDEX2).^42^ Moreover, our study tracked adherence to the physiotherapy program online and only persons who adhered were considered in the analyses. Adherence is assessed and collected by the JA app based on participants completing their allocated exercises and educational sessions and is calculated as a percentage of online sessions attended by each research participant. In the randomized controlled trial, iBEAT-OA, the mean adherence was 87.9% (SD 14.3%) of sessions completed.^21^ Other strengths include the isokinetic muscle strength testing measurements and metrologist reliably assessed function (walking speed, chair stands). The availability of IL-6 serum levels in addition to these traits has made it possible to link changes in microbiome to systemic inflammation.

In conclusion, 6 weeks of physiotherapy for knee pain that resulted in improved function and muscle strength had modest but significant effects on gut microbiome composition. Future work could focus on exploring longitudinal changes beyond the 6-week intervention period to assess the durability of the observed microbiome alterations and their long-term implications for muscle strength and inflammation. In addition, future work could focus on exploring synergistic effects of physiotherapy combined with microbiome-targeted interventions, such as fiber supplementation, in persons with chronic pain.

## Supplemental Material

sj-pdf-1-sph-10.1177_19417381241283812 – Supplemental material for Physical Therapy for Knee Pain Relief Induces Changes in Gut Microbiome Composition: A Secondary Analysis of Data From a Randomized Controlled Trial Supplemental material, sj-pdf-1-sph-10.1177_19417381241283812 for Physical Therapy for Knee Pain Relief Induces Changes in Gut Microbiome Composition: A Secondary Analysis of Data From a Randomized Controlled Trial by Afroditi Kouraki, Amrita Vijay, Sameer Gohir, Bonnie Millar, Anthony Kelly and Ana M Valdes in Sports Health
