# Supplementary material for: Physical Therapy for Knee Pain Relief Induces Changes in Gut Microbiome Composition: A Secondary Analysis of Data From a Randomized Controlled Trial
Source: Sports Health. 2024 Oct 6;17(5):1053–62. doi: 10.1177/19417381241283812 (PMC11556638; doi:10.1177/19417381241283812)
Supplement: sj-pdf-1-sph-10.1177_19417381241283812 – Supplemental material for Physical Therapy for Knee Pain Relief Induces Changes in Gut Microbiome Composition: A Secondary Analysis of Data From a Randomized Controlled Trial [file sj-pdf-1-sph-10.1177_19417381241283812.pdf]

**ONLINE APPENDIX**

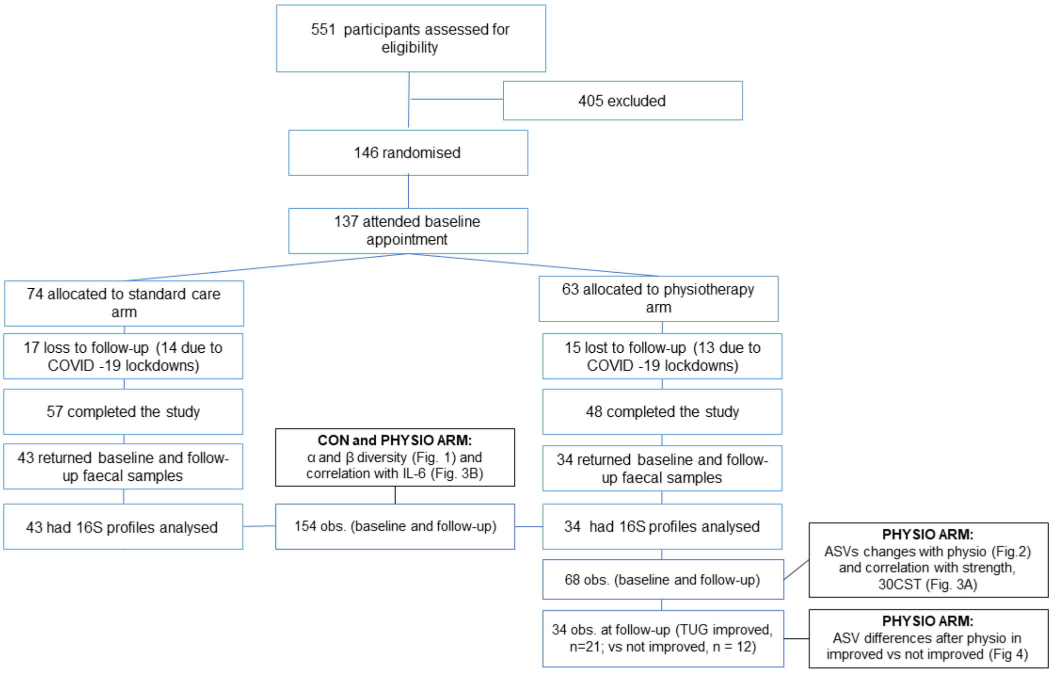


Fig. 1 Flow diagram of participants' recruitment, group allocation, the selection of participants included in the secondary analysis (i.e. those with available gut microbiome composition), and the number of observations used for each analysis

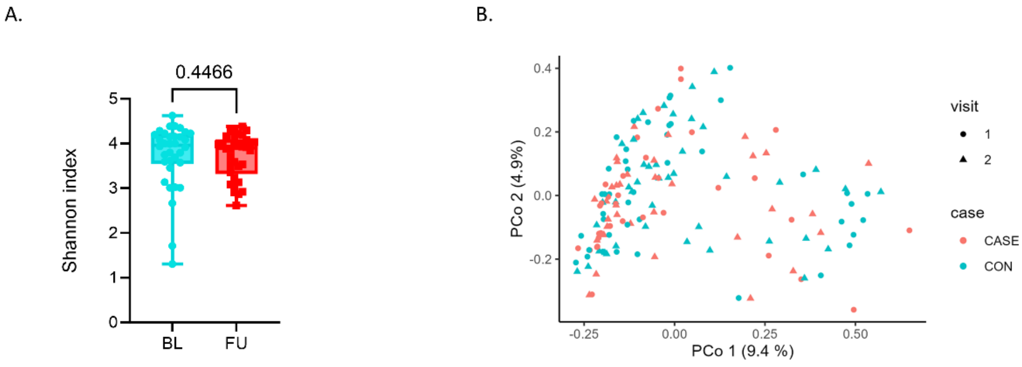


Fig.2 Comparisons of α diversity (Shannon index) and β diversity from baseline to follow-up: a. boxplots of Shannon from baseline (blue) to follow-up (red) in the active (physiotherapy) group, and b. PCoA of Bray Curtis distance from baseline (circles) to follow-up (triangles), visualising microbial composition in active (red) and control (light blue).

## Supplementary methods

**An overview of the ALDEX2 method and the steps it performs:**

The ALDEX2 method is a statistical analysis method used in microbiome data analysis. It is designed to identify differentially abundant taxa (microbial species) between different experimental conditions. Here are the detailed steps that the ALDEX2 method performs:

1. Generation of Posterior Probabilities:
    - ALDEX2 starts by generating posterior probabilities for each taxon (microbial species). These probabilities are obtained through Monte-Carlo sampling from a Dirichlet distribution with a small non-zero prior. This step helps handle zero values and accounts for the total read count to improve precision. The number of samples (n=128) specifies how many Monte-Carlo samples are generated for each taxon.
2. Centered Log-Ratio Transformation:
    - Once the posterior probabilities are calculated, ALDEX2 applies the centered log-ratio (CLR) transformation to each taxon's abundance profile. The CLR transformation is a common technique used to address compositional data in microbiome analysis. It helps to mitigate the effects of compositional constraints and makes the data more amenable to statistical analysis.
3. Statistical Testing:
    - ALDEX2 uses two different statistical tests, Welch's t-test and the Wilcoxon rank test, for each taxon in every simulated instance. These tests are applied to assess whether the differences in taxon abundance between experimental conditions are statistically significant.
4. Effect Size Calculation:
    - After conducting the statistical tests for each taxon in multiple instances (based on Monte-Carlo sampling), ALDEX2 calculates the effect size. The effect size is determined as the difference in taxon abundance between conditions divided by the maximum difference within conditions. This provides a standardized measure of effect size by scaling between-group differences with maximum within-group differences. Effect sizes can help interpret the practical significance of observed differences.
5. Calculation of Expected P-Values:

- Finally, ALDEX2 obtains expected p-values for each taxon by averaging over all instances. These expected p-values provide a more robust and reliable assessment of the statistical significance of differential abundance between conditions, accounting for the variability introduced by Monte-Carlo sampling.

In summary, the ALDEX2 method combines probabilistic modeling, transformation of data, statistical testing, effect size calculation, and p-value averaging to identify differentially abundant taxa in microbiome datasets while addressing issues such as zero values and compositional data constraints.

**Details on the use of ALDEX2 within the present study**

We used ADLEX2 to identify differences in ASVs between those who improved their quadriceps and hamstring muscle strength, 30-CST and TUG following the physiotherapy intervention. We used only the follow-up abundance data for the differential expression analysis as ALDEX2 cannot handle negative values and due to the limited sample size available only ASVs that were present in at least 50% of all samples and which had non-zero or non-near zero variance for change in abundance and for the trait under consideration were included in the analysis. This reduced the number of ASVs included to n=34.

**An overview of PERMANOVA:**

PERMANOVA is a statistical method used in ecology and bioinformatics to analyse patterns of dissimilarity among groups of samples, such as those obtained from metagenomic data and operates by testing the significance of differences in multivariate dispersion between groups of samples assessing whether the multivariate means or “centroids” of groups of samples are different, whilst taking into account the dispersion around the centroids

**Details on the use of PERMANOVA within the present study**

Because the microbiome samples were collected at two time points, we assessed differences in Bray Curtis between intervention and control using PERMANOVA performed with the ‘adonis’ function in R’s vegan package accounting for repeated measures. The change data were plotted in PCoA to avoid duplication.
